# Rat hepatic stellate cells alter the gene expression profile and promote the growth, migration and invasion of hepatocellular carcinoma cells

**DOI:** 10.3892/mmr.2014.2435

**Published:** 2014-07-31

**Authors:** ZHI-MING WANG, LE-YUAN ZHOU, BIN-BIN LIU, QIN-AN JIA, YIN-YING DONG, YUN-HONG XIA, SHENG-LONG YE

**Affiliations:** 1 Liver Cancer Institute, Zhongshan Hospital, Fudan University, Shanghai, P.R. China; 2 Key Laboratory of Carcinogenesis and Cancer Invasion, Ministry of Education, Fudan University, Shanghai, P.R. China; 3 Department of Radiation Oncology, Zhongshan Hospital, Fudan University, Shanghai, P.R. China

**Keywords:** HSCs, hepatocellular carcinoma, cDNA microarray, matrix metalloprotein-2,-9

## Abstract

The aim of the present study was to examine the effects of activated hepatic stellate cells (HSCs) and their paracrine secretions, on hepatocellular cancer cell growth and gene expression *in vitro* and *in vivo*. Differentially expressed genes in McA-RH7777 hepatocellular carcinoma (HCC) cells following non-contact co-culture with activated stellate cells, were identified by a cDNA microarray. The effect of the co-injection of HCC cells and activated HSCs on tumor size in rats was also investigated. Non-contact co-culture altered the expression of 573 HCC genes by >2-fold of the control levels. Among the six selected genes, ELISA revealed increased protein levels of hepatic growth factor, matrix metalloproteinase-2 (MMP-2) and −9 (MMP-9). Incubation of HCC cells with medium conditioned by activated HSCs significantly increased the proliferation rate (P<0.001), migration rate and the number of invasive HCC cells (P=0.001). Co-injection of HCC cells and activated HSCs into rats significantly increased the weight of the resulting HCC tumors (P<0.01). The paracrine activity of activated HSCs markedly altered the gene expression profile of HCC cells and affected their growth, migration and invasiveness. The results from the present study indicate that the interaction between the activated HSCs and HCC has an important role in the development of HCC.

## Introduction

Hepatocellular carcinoma (HCC) is a highly prevalent malignancy that is the third leading cause of cancer-related mortality worldwide (1). Although significant progress has been made in understanding the cellular and molecular biology of HCC, the prognosis for HCC patients remains poor. In China, the majority of HCC cases are secondary to hepatitis B and C, and are often accompanied by hepatic fibrosis/cirrhosis. Fibrosis occurs in response to hepatocyte injury and the subsequent activation of hepatic stellate cells (HSCs) (2). The combined effects of hepatocyte injury and HSC activation are required for the development of hepatic fibrosis/cirrhosis. Hepatic cirrhosis enhances hepatocyte sensitivity to carcinogenic factors in the environment and to liver cancer associated pro-inflammatory cytokines. Hepatitis, hepatic fibrosis/cirrhosis and liver cancer are thus regarded as the ‘inflammation-fibrosis-cancer axis’ in the pathogenesis of liver carcinoma (3,4).

Activated HSCs are a major component of the liver interstitium in HCC. Chau *et al* (5) investigated HCC liver tissue using immunohistochemistry and electron microscopy, and identified numerous myofibroblasts (activated HSCs) among the cancer cells. Activated HSCs also secrete large quantities of collagen, providing a collagen source for HCC capsule formation and the accumulation of excess collagen in HCCs without a capsule (6,7).

Several studies have confirmed that activated HSCs promote the proliferation and development of HCC cells *in vitro* and *in vivo* (8,9). However, the mechanism by which activated HSCs affect HCC cells remains elusive. In the present study, we examined whether activated HSCs induce specific changes in HCC gene expression, and began preliminary investigations into the resulting differences in protein expression. In addition, the effects of activated HSCs on the proliferation, migration, and invasiveness of HCCs was investigated *in vitro* and on tumor growth *in vivo*.

## Materials and methods

### Animals

Specific pathogen-free Buffalo rats (male and female) were purchased from the Charles River Laboratory (Wilmington, MA, USA) and maintained at the Shanghai Laboratory Animals Co., Ltd. (SLAC; Shanghai, China). Male Buffalo rats weighing 200–250 g were provided by SLAC and housed in the Experimental Animal Center of Affiliated Zhongshan Hospital of Fudan University (Shanghai, China). Animals were provided access to water and food *ad libitum*. The present study was approved by the Ethics Committee of the Affiliated Zhongshan Hospital of Fudan University.

### Cell lines

McA-RH7777 liver cancer cells from Buffalo rats were purchased from American Type Culture Collection (Mannassas, VA, USA).

### McA-RH7777/hepatic stellate cell co-culture and examination

#### Preparation of liver cancer cell-conditioned medium (CM)

McA-RH7777 liver cancer cells were maintained in DMEM containing 10% fetal bovine serum (FBS) at 37°C in an environment with 5% CO_2_. As described in previous studies (10), when the cells were ~100% confluent, they were washed and maintained in serum-free DMEM for 2 h. This medium was then discarded and the cells were incubated in serum-free DMEM for 24 h. The medium from the second incubation was obtained and centrifuged, and the supernatant was collected, filtered through a 0.22 μm filter and stored at −20°C until use.

#### Preparation of induction-activated HSCs (iHSCs)

HSCs were separated from the rat liver using a modified Friedman method (11). Newly separated HSCs were maintained in DMEM containing 10% FBS for two days and then in McA-RH7777-CM (50% CM, 10% FBS and 40% DMEM) for 5–7 days to produce iHSCs (10). Several methods were employed to determine the purity of HSCs. The freshly separated cells were observed and identified. Under a light microscope, lipid droplets have high refractivity and spontaneous fluorescence. Following two days in culture, the purity of the HSCs was evaluated based on autofluorescence intensity and the fluorescence intensity of desmin-positive cells. HSC purity was demonstrated to be >95%. Immunofluorescence staining of alpha-smooth muscle actin (α-SMA) was used to detect activated HSCs.

#### HCC cell culture

McA-RH7777 cells in the exponential growth phase were digested with trypsin solution containing EDTA and then suspended in DMEM containing 10% FBS at a cell density of 2×10^5^/ml. In the control group, McA-RH7777 cells (2 ml/well) were cultured alone, while in the experimental group, the cells were co-cultured with iHSCs in a transwell chamber (diameter, 24 mm; pore size, 0.4 μm; Corning, Shanghai, China). McA-RH7777 cells were seeded into the lower chamber (cell density similar to that of the controls) and the iHSC suspension (1 ml/well) was added to the upper chamber (2×10^5^/ml). McA-RH7777 cells or iHSCs were maintained in DMEM containing 10% FBS for 24 h and then in DMEM containing 1% FBS for three days.

#### cDNA microarray analysis

Total RNA extracted from the control McA-RH7777 cells cultured alone or co-cultured with iHSCs was analyzed using a cDNA microarray. Two independent isolations and microarray analyses (experiments 1 and 2) were performed using 4×44K Rat Genome Array chips (Agilent Technologies, Santa Clara, CA, USA) according to the manufacturer’s instructions. Data analysis was performed using Feature Extraction and GeneSpring 10.0 software (Agilent Technologies).

#### Quantitative (q)PCR analysis of gene expression

To quantify selected gene expression, McA-RH7777 cells or McA-RH7777 cells co-cultured with iHSCs were lysed with TRIzol reagent (Invitrogen Life Technologies, Carlsbad, CA, USA) at a concentration of 100 μl/10^6^ cells and the total RNA was extracted. The mRNA was reverse-transcribed using an oligo (dT) 18–25 primer and Omniscript reverse transcriptase (Toyobo, Co., Ltd., Osaka, Japan). The selected gene transcripts were quantified by qPCR using gene-specific primers (Table I) and SYBR Green Realtime PCR Master Mix (Toyobo, Co., Ltd.). The expression of glyceraldehyde 3-phosphate dehydrogenase (GADPH) was used as an internal control. PCR amplification conditions were as follows: 95°C for 10 sec, followed by 40 cycles of 95°C for 5 sec and 60°C for 20 sec. Samples were run in triplicate using a real-time PCR thermocycler (ABI PRISM 7000 Sequence Detection System; Applied Biosystems, Foster City, CA, USA) and the results were analyzed using matched software. Relative gene expression was determined by normalizing to GAPDH expression in each set of the samples according to the manufacturer’s instructions. According to the sequences of the target gene and GAPDH in GenBank, the corresponding primers were designed using Premier 5.0 and BLAST was used to exclude the homologous sequences (Table II).

#### Western blot analysis of interferon regulatory proteins and integrin αE

McA-RH7777 cells (1×10^6^) cultured alone or co-cultured with iHSCs were lysed with 1 ml RIPA buffer containing 10 μl PMSF. Total protein was prepared by standard procedures. The total protein concentration was estimated by the Bradford assay, with BSA as the standard. The proteins were separated by SDS-PAGE and transferred to polyvinylidene difluoride membranes. Membranes were blocked for 1 h at room temperature in 5% non-fat milk in 0.1% Tween-20, then washed and incubated overnight at 4°C with an anti-rat antibody against one of the following proteins: interferon regulatory factor 1 (Irf1; Santa Cruz Biotechnology, Inc., Santa Cruz, CA, USA), interferon regulatory factor 9 (Irf9; Santa Cruz Biotechnology, Inc.), integrin alpha E (1:1500; ITGAE; Biolegend, San Diego, CA, USA) or β-actin (1:1000; BD Pharmingen, San Jose, CA, USA). Immunodetection was performed using the ECL blotting detection system (Bio-Rad, Hercules, CA, USA).

### McA-RH777 cells incubated with iHSC-CM

#### Preparation of iHSC-CM (iHSC-CM)

Newly separated HSCs were maintained in DMEM containing 10% FBS for two days and then in CM-containing medium (50% CM, 10% FBS and 40% DMEM) for 5–7 days to induce activation of the cells. The medium was then refreshed and the cells maintained in serum-free DMEM for 24 h, following which the supernatant was collected, centrifuged and used as iHSC-CM.

#### HCC cell proliferation assay

McA-RH7777 cells in the exponential growth phase were digested with trypsin and re-suspended in DMEM containing 20% FBS (1×10^5^/ml). These cells were seeded into 96-well plates (50 μl/well) and divided into two groups (6 wells/group). In the treatment group, 50 μl of iHSC-CM was added to each well, while in the control group, 50 μl of DMEM was added to each well. Cell proliferation was detected with CCK-8 (Cell counting kit-8; Nanjing KeyGen Biotech Co., Ltd., Nanjing, China) according to the manufacturer’s instructions.

#### HCC migration assay (scratch test)

McA-RH7777 cells were digested with trypsin and suspended in DMEM containing 10% FBS (2×10^5^/ml). These cells were then seeded onto 6-well plates (3 ml/well). When cells reached 70% confluence, they were maintained in serum-free medium for 12 h and then divided into two groups (3 wells/group). The supernatant was removed and the cell layer was scratched with a 10-μl pipette tip. Cells were then washed with PBS to remove shedding cells. In the experimental group, cells were maintained in DMEM (1.5 ml) containing 2% FBS and 1.5 ml of iHSC-CM. In the control group, cells were grown in DMEM (3 ml) containing 1% FBS. Cell migration was observed under a light microscope (DFC500; Leica, Wetzlar, Germany).

#### Detection of HCC invasion

A transwell chamber was prepared by adding Matrigel to the chamber, followed by incubation at 4°C overnight. Serum-free DMEM was diluted at a ratio of 1:7 and then added to the upper chamber (80 μl). The transwell chamber was placed on a plate and incubated at 37°C for 12 h. A suspension of McA-RH7777 cells with a density of 1×10^5^ cells/ml was prepared. In the control group, cells were suspended in serum-free DMEM, while in the experimental group, cells were suspended in a solution with a 1:1 ratio of serum-free DMEM and iHSC-CM. The cell density in the two groups was identical. These cells (200 μl) were added to the upper chamber of the transwell chamber. In the control group, DMEM containing 10% FBS (600 μl) was added to the lower chamber. In the experimental group, DMEM containing 20% FBS (300 μl) and iHSC-CM (300 μl) was added to the lower chamber. Cells were then incubated for 36 h and fixed in neutral formalin. The Matrigel and cells in upper chamber were removed, followed by Giemsa staining. Several fields were randomly selected under a light microscope followed by cell counting to determine the extent of invasion.

### Changes in protein expression in co-cultured cells

McA-RH7777 cells in the exponential growth phase were digested with trypsin and suspended in DMEM containing 10% FBS at a density of 4×10^5^/ml. These cells were seeded into 6-well plates (2 ml/well) and divided into two groups (3 wells/group). In the control group, McA-RH7777 cells were cultured alone, while in the experimental group, cells were co-cultured with iHSCs in a transwell chamber (diameter, 24 mm; pore size, 0.4 μm). McA-RH7777 cells were seeded into the lower chamber (cell density was similar to that of controls) and iHSC suspension (1 ml) was added to the upper chamber (2×10^5^/ml). One day later, the medium was refreshed with serum-free DMEM and incubated for two days. The supernatant was then collected from the two groups and ELISA (R&D Labs, Minneapolis, MN, USA) was performed to detect the hepatic growth factor (HGF), interleukin 6 (IL-6), matrix metalloprotein-2 (MMP-2), matrix metalloprotein-9 (MMP-9), tumor necrosis factor-α (TNF-α) and transforming growth factor-β1 (TGF-β1).

### In vivo study of tumorigenicity

Rats were divided into two groups (n=6/group). In the control group, 200 μl of McA-RH7777 cells (2×10^6^ cells) were subcutaneously inoculated into the limbs of Buffalo rats. In the experimental group, 200 μl of McA-RH7777 cells (2×10^6^ cells) and 200 μl of iHSC (1×10^6^ cells) were subcutaneously inoculated into the limbs of these rats. Three weeks following inoculation, rats were sacrificed with an anesthesia overdose and the subcutaneous tumor was collected, weighed, embedded in paraffin and sectioned, followed by H&E staining. The dimensions of the tumor were then recorded.

### Statistical analysis

Data were presented as the mean ± standard deviation. Two-sided values of P<0.05 were considered to indicate a statistically significant result. Differences in the quantitative data between McA-RH7777+iHSC and the control groups were examined using the independent two samples t-test. Statistical analyses were performed using SPSS 15.0 statistics software (SPSS, Inc, Chicago, IL, USA).

## Results

### Identification of HSCs and iHSCs

**Figure 1:**
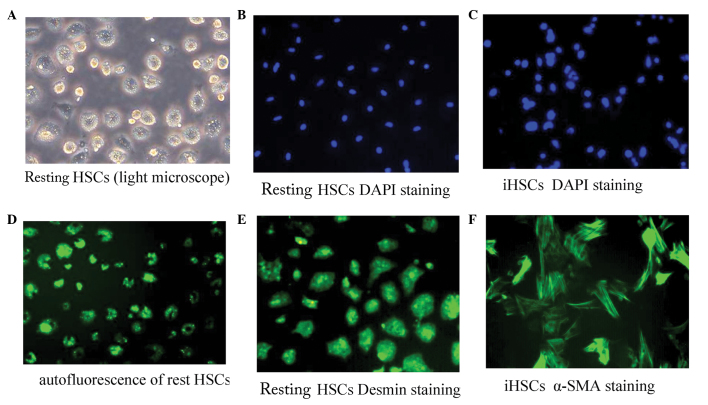
(A) Refraction of lipid droplets in resting HSCs observed under light microscopy. (B) DAPI staining of nuclei in resting HSCs. (C) DAPI staining of iHSC nuclei. (D) Blue-green fluorescence in resting HSCs following excitation at 328 nm. (E) Desmin staining demonstrated the purity of HSCs was >95%. (F) α-SMA positive iHSCs. HSCs, hepatic stellate cells; iHSCs, induction-activated HSCs.

Fig. 1 demonstrates the histological characteristics of resting and activated HSCs. In resting HSCs, refraction of intracellular lipid droplets (Fig. 1A) and blue-green fluorescence following excitation at 328 nm (Fig. 1B) were observed. Desmin staining demonstrated the purity of HSCs to be >95%. HSCs activated by incubation with HCC-CM revealed positive staining for α-smooth muscle actin (α-SMA), a marker for activated cells (Fig. 1F).

### Co-culture with iHSCs alters gene expression in McA-RH7777 cells via a paracrine mechanism

#### Microarray results

**Figure 2:**
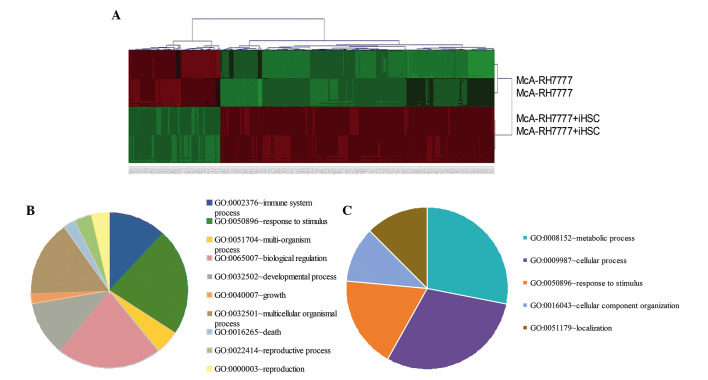
(A) Genes upregulated (red) or downregulated (green) >2-fold in McA-RH7777 cells by co-culture with iHSCs. (B) Classification of the 432 upregulated genes into ten groups by GO analysis. (C) Classification of the 141 downregulated genes into five groups by GO analysis. HSCs, hepatic stellate cells; iHSCs, induction-activated HSCs.

cDNA microarray examination detected 28,728 genes, 573 of which were up- or downregulated >2-fold over the control levels (HCC cells cultured alone). Of the genes with altered expression levels, 432 were upregulated and 141 were downregulated (Fig. 2A). Upregulated genes include Tapbp, Ccl2, Cxcl1, Cxcl10, Junb, Igf1, Stat1, Irf7, Irf9 and Csf1. 4-Sep was among the downregulated genes. Based on GO enrichment analysis, upregulated genes were classified into ten groups (Fig. 2B) and downregulated genes were classified into five groups (Fig. 2C). The genes with altered expression encoded cell surface receptors, proteins involved in cell metabolism, cell adhesion molecules, cell signaling pathway molecules, chemokines and immune-associated factors.

#### Confirmation of cDNA microarray assay results by qPCR

**Figure 3:**
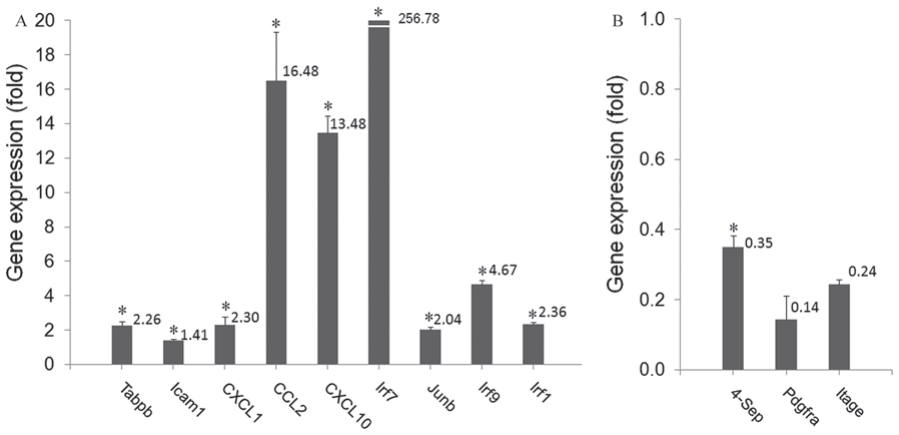
qPCR of genes with altered expression levels, as identified by microarray analysis. (A) Expression levels of selected McA-RH7777 cell genes upregulated in the presence of iHSCs. (B) Expression levels of selected McA-RH7777 genes downregulated in the presence of iHSCs. Data are presented as the mean ± standard deviation. n=3 in each group; ^*^P<0.05, compared with the control. qPCR, quantitative PCR; iHSCs, induction-activated hepatic stellate cells.

qPCR was used to detect the expression of selected genes, to validate the findings from the cDNA microarray assay. The results demonstrated a significant increase in the expression of Tabpb, Icam1, Cxcl1, Ccl2, CXCL10, Junb, Irf7, Irf9 and Irf1 (Fig. 3A) in the McA-RH7777-iHSC cells compared with the controls (range, 1.4–256. 8-fold; P≤0.016). The expression level for 4-Sep (Fig. 3B) decreased significantly compared with the controls (0.35 fold; P<0.001). The expression levels of PDGFRA and ITGAE also decreased, however these differences did not reach statistical significance (P>0.05). These qPCR findings confirmed the results of the cDNA microarray assay.

#### Confirmation by western blot analysis

**Figure 4:**
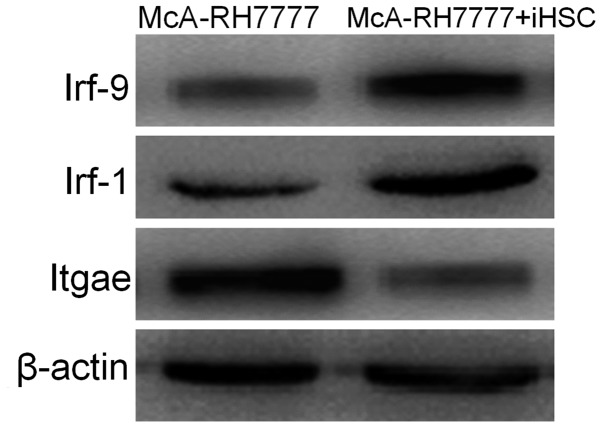
Western blot analysis demonstrating that in McA-RH7777 cells co-cultured with iHSCs, the ITGAE gene was downregulated, and the Irf1 and Irf9 genes were upregulated. iHSCs, induction-activated hepatic stellate cells.

Western blot analysis was used to detect the protein expression of three genes. Co-culture of McA-RH7777 with iHSC downregulated the expression of integrin αE protein and upregulated the protein expression of Irf1 and Irf9 as compared with the protein expression levels in McA-RH7777 control cells. These results were consistent with those of the qPCR and cDNA microarray assays (Fig. 4).

#### iHSCs promote proliferation, migration and invasion of HCC cells via paracrine mechanisms

**Figure 5:**
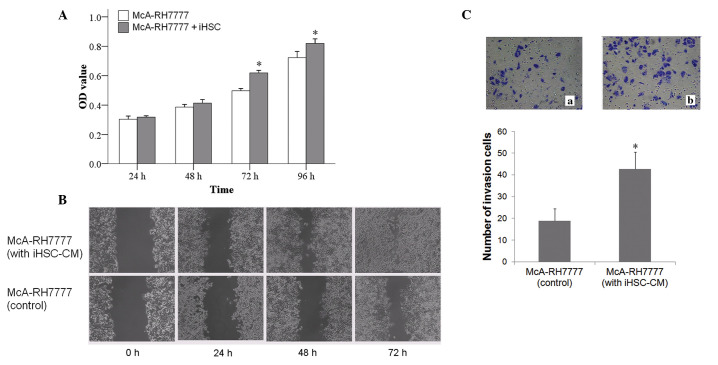
(A) CCK-8 assay demonstrating iHSC-CM promotion of the proliferation of HCC cells (n=6 in each group; ^*^P<0.05). (B) Scratch test demonstrating that CM from iHSCs increased the migration of HCC cells. (C) Transwell assay demonstrating that the number of migrated cells increased significantly when HCC cells were incubated with iHSC-CM and that iHSC-CM promoted the invasion of HCC cells. In the experimental group (McA-RH7777 + iHSC-CM) and the control group (McA-RH7777), the number of migrated cells was 42.5±7.9 and 18.8±5.5, respectively, demonstrating a significant difference (n=5 in each group; P<0.05). ^*^ P<0.05, compared with the control. Quantitative data are presented as the mean ± standard deviation. OD, optical density; CCK-8, cell counting kit-8; iHSCs, induction-activated HSCs; CM, conditioned medium; HCC, hepatocellular carcinoma.

CM from iHSCs promotes HCC cell proliferation. In the presence of iHSC-CM, HCC cells proliferated more rapidly than the control cells. Increased cell counts were observed in the McA-RH7777 and the McA-RH7777 + iHSC groups. However the cell count [Optical density (OD) value)] increased more rapidly in the McA-RH7777 + iHSC co-culture than in McA-RH7777 alone. This cell count difference reached significance at 72 h (OD value, 0.62 vs. 0.50; P<0.001) and remained at 96 h (OD value, 0.82 vs. 0.72; P=0.001; Fig. 5A).

#### CM from iHSCs promotes migration of HCC cells

To investigate the effect of iHSC paracrine mechanisms on the migration of HCC cells, the scratch test was employed. In the presence of iHSC-CM, the migration rate of HCC cells increased markedly (Fig. 5B).

#### CM of iHSCs increased the invasiveness of HCC cells

The transwell assay demonstrated that the number of invasive cells in the McA-RH7777 + iHSC-CM preparation was significantly higher than in the controls (42.6 vs. 18.8, P=0.001; Fig. 5C).

### Co-culture induces changes in protein secretion from HCC cells

**Figure 6:**
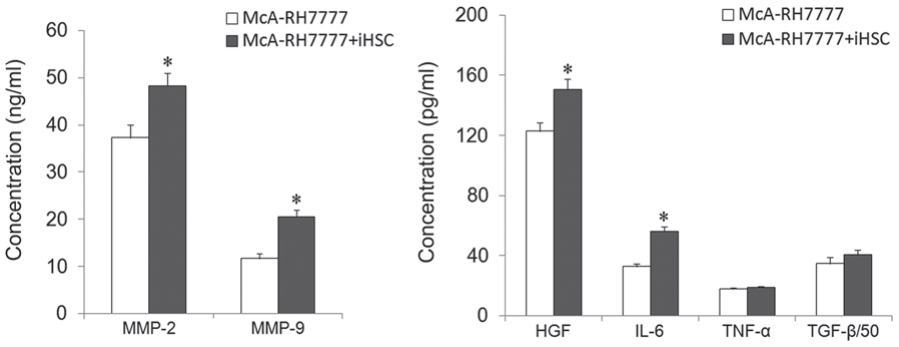
Comparison of cytokines in the supernatant. The units of MMP-2 and MMP-9 concentrations are ng/ml and the units for the other parameters are pg/ml. TGF-β1 was measured following 50-fold dilution. Data are presented as the mean ± standard deviation. n=3 in each group. ^*^P<0.05, compared with the control. MMP, matrix metalloproteinase; IL-6, interleukin-6; TNF-α, tumor necrosis factor-α; TGF-β1, transforming growth factor-β1.

ELISA of cell culture medium revealed that the concentrations of HGF (150.4 vs. 122.8 ng/ml), IL-6 (56.3 vs. 32.8 ng/ml), MMP-2 (48.3 vs. 37.3 ng/ml) and MMP-9 (20.6 vs. 11.7 ng/ml) in the McA-RH7777 + iHSC group were significantly higher than in the control group (all P≤0.007; Fig. 6). No significant difference was observed in TNF-α and TGF-β concentrations between McA-RH7777 + iHSC and the control groups.

### Tumorigenicity test in Buffalo rats

**Figure 7:**
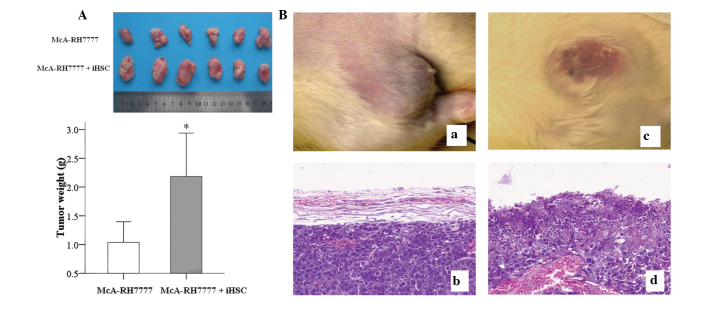
(A) Proliferation of subcutaneous cancer cells. Mean tumor weight was 1.04±0.36 g in group A and 2.18±0.75 g in group B, which was a significant difference (P<0.01). (B) iHSCs increased the tumorigenicity of McA-RH7777 cells; (Ba) rats were inoculated with McA-RH7777 cells alone and the cancer did not invade the skin; (Bb) pathological examination (H&E staining) confirmed that the cancer had a complete capsule; (Bc) rats were inoculated with McA-RH7777 cells and iHSCs and (Bd) pathological examination (H&E staining) confirmed that the cancer had invaded the skin. iHSCs, induction-activated hepatic stellate cells.

By three weeks following inoculation, the cancer cells had invaded the skin of rats co-injected with McA-RH777 cells and iHSCs. Three of the six rats developed skin ulcers and skin invasion was pathologically confirmed in all three rats. However, in the rats injected with HCC cells alone, the skin was not involved and the cancers had complete capsules. The tumors weighed significantly more in the McA-RH7777 + iHSC group than in controls (2.18 vs. 1.04 g; P<0.01; Fig. 7).

## Discussion

In the present study, we observed that the incubation of HCC cells in iHSC-CM increased the rate of HCC cell proliferation and migration, and the number of invasive HCC cells. Co-culture of iHSCs and HCC cells induced extensive changes in the gene expression profile and increased the expression of HGF, IL-6, MMP-2 and MMP-9 in HCC cells. In addition, co-injection of HCC and activated HSCs into rats significantly increased the weight of the resulting HCC tumors.

HSCs are located in Disse’s space and are rich in cytoplasmic lipid droplets containing vitamin A. HSCs account for ~3% of non-parenchymal cells (12). In the presence of liver injury, HSCs become activated and transform into myofibroblasts. These activated HSCs are characterized by changes in morphology, a reduction in lipid droplets, the presence of α-SMA and active proliferation (13,14). As observed in the current study, these activated HSCs have been demonstrated to increase the growth and proliferation of HCCs *in vitro* and *in vivo* (9,15).

Previously, we demonstrated that treatment of stellate cells with HCC-CM resulted in a gene expression profile that differed from that observed in stellate cells activated by culture medium alone (10). In the present study, the converse experiment was conducted. The co-culture of activated stellate cells and HCC cells under non-contact conditions was observed to alter the gene expression profile of the HCC cells. Thus, the stellate cells secreted substances that triggered gene expression alterations in the HCC cells.

Results of the microarray assay demonstrated changes in the expression of 573 HCC cell genes following co-culture with stellate cells. There are numerous known functions of these genes, including as cell surface receptors, proteins which involved in cell metabolism, adhesion molecules, signaling pathway molecules, chemokines and immune associated factors. These genes have functions similar to those with altered expression in HSCs activated by HCC-CM. We therefore hypothesized that iHSCs may have an extensive effects on McA-RH7777 HCC cells by impacting numerousgenes involved in cell survival, proliferation, metabolism and immunity. The extent to which these changes in gene expression actually alter protein expression are not addressed in the present study and require further investigation.

The present study also investigated the effects of iHSC-CM on the proliferation, migration and invasiveness of HCC cells. It was observed that iHSC-CM promoted the proliferation, migration and invasion of HCC cells *in vitro*, a result consistent with previous studies (9,15,16). Our *in vivo* results were similar. The tumorigenicity test demonstrated that cancer occurred earlier in HCC cells co-inoculated with iHSCs. Four days following co-inoculation, a rice-sized mass was identified under the skin in the co-injected rats. However, in the group inoculated with HCC cells alone, such a mass was not identified until one week after inoculation. This *in vivo* evidence further confirmed that iHSCs promoted the growth and invasion of HCC cells, similar to results reported by Amann *et al* (15).

The possible mechanism underlying the paracrine effects of iHSCs was investigated by examining the expression of key regulatory proteins in HCC cells in response to iHSC paracrine factors. TGF-β has been demonstrated to be increased in liver injury and to be important in activating HSCs and transforming them into myofibroblasts (17). However, HCC cells co-cultured with iHSCs demonstrated no change in TGF-β1 or TNF-α production. These results were unexpected given the known activities of TGF-β. We suggested that while TGF-β may promote HSC activation, the HSC-induced changes observed here were independent of TGF-β activity.

Our preliminary protein studies investigated the expression levels of proteins encoded by six of the genes overexpressed in HCC cells following co-culture with iHSCs. Marked increases in the expression of HGF, IL-6, MMP-2 and MMP-9 were observed (P<0.05). HGF promotes the endothelial mesenchymal transition of HCC cells and cancer formation, and has been reported to act through the Akt pathway (18). However, HCC migration in the presence of HSCs has been reported by Santomoto *et al* to depend on the MEK/ERK pathway and not on the P13K/Akt pathway (19). IL-6 may facilitate angiogenesis, vascularization and tumorigenicity (20), and may have an important role in tumor progression (21). MMPs degrade the extracellular matrix and have been demonstrated to promote the invasion and metastasis of cancer cells (22). Therefore, the increase in MMP-2 and MMP-9 observed in our study may be associated with the increase invasiveness of HCC cells following exposure to iHSCs. Evidence from other studies on the mechanisms underlying how iHSCs increase HCC growth and invasiveness vary. The NFκB and ERK pathways have been reported by a number of studies to be involved (15,19). In addition, HSCs have been reported to have a systemic immunosuppressive effect, and to promote angiogenesis and proliferation (9).

In summary, the *in vitro* alterations in the gene expression profile of HCC cells in response to co-cultivation with iHSCs were extensive. While the significance of changes in the expression of specific genes has not been addressed here, we hypothesize that the interaction between HSCs and HCC cells *in vivo* may be multifaceted, and that activated HSCs may have an important role in the progression of HCC. Alterations in the expression of a number of the genes observed in our microarray results may be involved in the regulation of invasion/metastasis and may contribute to the accelerated tumor growth noted in the *in vivo* experiments. Investigation of these changes and of strategies to inhibit them may have considerable clinical applications in the future.

## Figures and Tables

**Table I:** PCR primer sequences.

Gene	Primer pairs	Accession no.
Ccl2	sense: AGTCGGCTGGAGAACTACAAG anti-sense: TGAAGTCCTTAGGGTTGATGC	NM_031530
Cxcl1	sense: ACCCAAACCGAAGTCATAGC anti-sense: GGGACACCCTTTAGCATCTT	NM_030845
Cxcl10	sense: CATGAACAGACGCTGAGACC anti-sense: TGCGGACAGGATAGACTTGC	NM_139089
Irf1	sense: TGAAGGACCAGAGCAGGAA anti-sense: GTCAGATAAGGTGTCAGGGCTA	NM_012591
Irf7	sense: TCTGGAGAACAGGGAAGAAGT anti-sense: GTGGCTGTATTGCAGAACCT	NM_001033691
Irf9	sense: GCCATTCAAGCGAAGTATCAG anti-sense: CCGCCATAGATGAAGGTGAG	NM_001012041
Icam1	sense: CAAACGGGAGATGAATGGT anti-sense: CTCTGGCGGTAATAGGTGTAA	NM_012967
Itgae	sense: CTGCCTTATGAAGTGGAGCG anti-sense: TGGAGATGAGCCCGAAGTGT	AF020046
Junb	sense: TAAAGAGGAACCGCAGACC anti-sense: GCTTTCGCTCCACTTTGAT	NM_021836
Pdgfra	sense: GCCAGGAGACGAGGTATCAA anti-sense: TCCCAGAGCAGAACGCCATA	M63837
Sept4	sense: CAAGTTGAGGACAATGCTGGTG anti-sense: GCGATTCCGTTCCTTCACTA	NM_001011893
Tapbp	sense: CTTGGGATGACGACAATGAT anti-sense: AATGGTGACGGACAGTGGAGAC	NM_033098
Gapdh	sense: TACAAGGAGTAAGAAACCGTGGAC anti-sense: GTTATTATGGGGTCTGGGATGG	NM_017008

GADPH, glyceraldehyde 3-phosphate dehydrogenase.

**Table II:** qPCR was performed to confirm the results from the DNA microarray assay.

		McA-RH7777		McA-RH7777 + iHSC		
				
Gene	Abbreviation	Exp. 1	Exp. 2	Exp. 1	Exp. 2	log2 iHSC^c^
TAP binding protein	Tapbp	9.609	9.868	11.842	11.797	2.081
Intercellular adhesion molecule 1	Icam1	12.916	12.918	14.14	14.053	1.1795
Chemokine (C-X-C motif) ligand 1^a^	Cxcl1	13.161	13.185	14.45	14.499	1.3015
Chemokine (C-C motif) ligand 2	Ccl2	4.617	4.38	6.42	6.411	1.917
Chemokine (C-X-C motif) ligand 10	Cxcl10	12.13	12.008	15.67	15.741	3.6365
Jun B proto-oncogene	Junb	9.242	9.274	10.43	10.415	1.1645
Interferon regulatory factor 7	Irf7	7.439	8.38	15.506	15.499	7.593
Interferon regulatory factor 9	Irf9	8.061	8.32	10.499	10.497	2.3075
Interferon regulatory factor 1	Irf1	6.754	7.285	9.867	9.843	2.8355
Septin 4	4-Sep	8.874	8.854	7.771	7.7	−1.1285
Platelet-derived growth factor receptor^b^	Pdgfra	6.51	7.079	5.499	5.458	−1.316
Integrin, alpha E	Itgae	11.244	11.021	10.081	10.021	−1.0815

a Melanoma growth stimulating activity, alpha;

b alpha polypeptide;

c vs. Control. Compared with McA-RH7777 cells cultured alone, Itgae expression was down-regulated, and the expression of Irf1 and Irf9 was up-regulated in McA-RH7777 cells co-cultured with iHSCs.

qPCR, quantitative PCR; Exp, experiment.
